# The role of nitric oxide in brain disorders: Autism spectrum disorder and other psychiatric, neurological, and neurodegenerative disorders

**DOI:** 10.1016/j.redox.2020.101567

**Published:** 2020-05-15

**Authors:** Manish Kumar Tripathi, Maryam Kartawy, Haitham Amal

**Affiliations:** Institute for Drug Research, School of Pharmacy, Faculty of Medicine, The Hebrew University of Jerusalem, Jerusalem, Israel

**Keywords:** Nitric oxide, S-nitrosylation, Autism spectrum disorder, Alzheimer's disease, Psychiatry, Neurodegeneration, Neurodevelopmental disorders, Brain disorders, SHANK3

## Abstract

Nitric oxide (NO) is a multifunctional signalling molecule and a neurotransmitter that plays an important role in physiological and pathophysiological processes. In physiological conditions, NO regulates cell survival, differentiation and proliferation of neurons. It also regulates synaptic activity, plasticity and vesicle trafficking. NO affects cellular signalling through protein S-nitrosylation, the NO-mediated posttranslational modification of cysteine thiols (SNO). SNO can affect protein activity, protein-protein interaction and protein localization. Numerous studies have shown that excessive NO and SNO can lead to nitrosative stress in the nervous system, contributing to neuropathology. In this review, we summarize the role of NO and SNO in the progression of neurodevelopmental, psychiatric and neurodegenerative disorders, with special attention to autism spectrum disorder (ASD). We provide mechanistic insights into the contribution of NO in diverse brain disorders. Finally, we suggest that pharmacological agents that can inhibit or augment the production of NO as well as new approaches to modulate the formation of SNO-proteins can serve as a promising approach for the treatment of diverse brain disorders.

## 1 Introduction

**Fig. 1:**
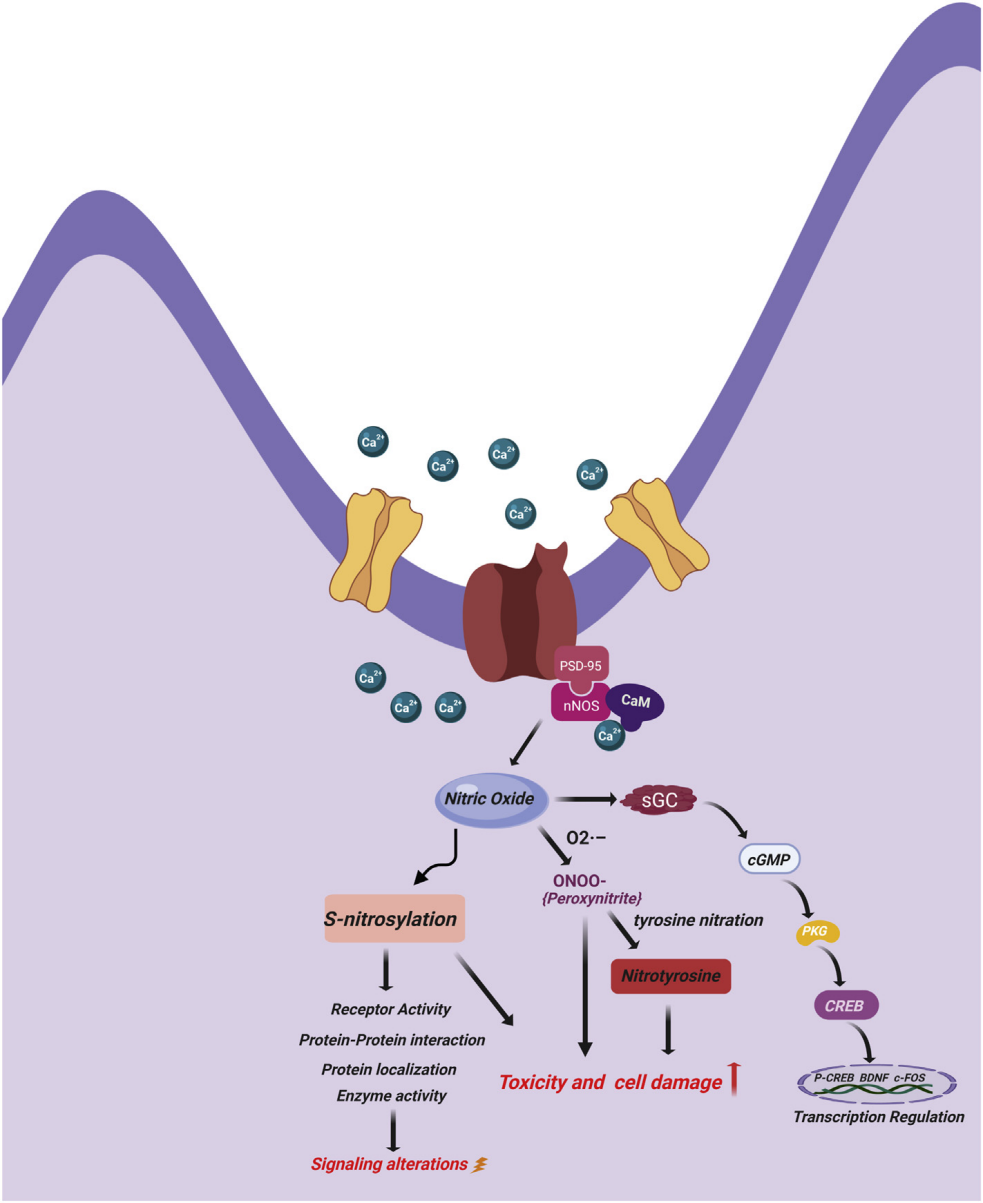
Schematic representation of NO signalling pathways in physiological conditions. Ca^+2^ influx activates nNOS by binding with calmodulin, leading to NO production. NO activates soluble guanylate cyclase to produce cGMP which interacts with many intracellular proteins such as PKG. PKG leads to CREB phosphorylation which leads into transcriptional activation of different genes. NO, directly and indirectly, leads to S-nitrosylation (SNO) of many proteins and receptors. SNO modification of proteins can alter the receptor activity, protein-protein interaction and protein localization leading to alteration in signalling. Increased level of NO increases nitrosative stress, proxynitrite formation, tyrosine nitration of proteins, which ultimately may lead into cell death.

Nitric oxide (NO) is one of the most important signalling molecules of the central nervous system (CNS) and peripheral nervous system (PNS) [[1], [2], [3]]. NO is produced in the brain from l-arginine by three nitric oxide synthase) isoforms (NOS1, NOS2, NOS3) [1]. Neuronal NOS (nNOS or NOS1) is constitutively expressed in the cytosol of neurons and requires Ca^2+^ for its activity. Inducible NOS (iNOS or NOS2) is found in the cytosol of glial cells and its activity is independent of Ca^2+^. Endothelial NOS (eNOS or NOS3) is constitutively expressed in endothelial cells in the membrane-bound state and requires Ca^2+^ for its activity [[4], [5], [6]]. nNOS is attached to *N*-methyl-d-aspartate receptor (NMDAR), post synaptic density protein-95 (PSD-95) and PSD-93 [7]. When the NMDAR gets activated by extracellular stimuli, it allows entry of Ca^2+^ inside the cell. Ca^2+^ can form a complex with calmodulin, and together they initiate the NO formation by activating NOS enzyme [8]. NO is a small gaseous molecule, which diffuses to activate guanosine monophosphate (GMP) cyclase (see Fig. 1). At low concentration, NO acts as a signalling molecule, taking part in the regulation of multiple functions in different organs and systems of the body. In the nervous system, it regulates synaptic activity, plasticity, and vesicle trafficking. However, at higher concentrations, NO may be toxic and could lead to cell death [9]. It reacts with superoxide radical (O_2^−^_) and forms peroxynitrite, which ultimately damages DNA, lipid and protein during oxidative stress [10]. NO may affect cellular signalling through proteins S-nitrosylation (SNO), tyrosine nitration, and S-nitrosoglutathione (GSNO) formation [[11], [12], [13], [14], [15]] (see Fig. 1). SNO is the NO-mediated posttranslational modification (PTM) of cysteine thiols, in which a nitrosogroup is incorporated into a reactive cysteine thiol and forms a nitrosothiol group [16,17]. SNO plays a role in protein localization, axonal transport, maintenance of synaptic plasticity and regulation of various neuronal pathways [9,18] (see Fig. 1). However, dysregulation of NO and SNO signalling is involved in progression of many neurodevelopmental, neurobehavioral and neurodegenerative disorders.

**Fig. 2:**
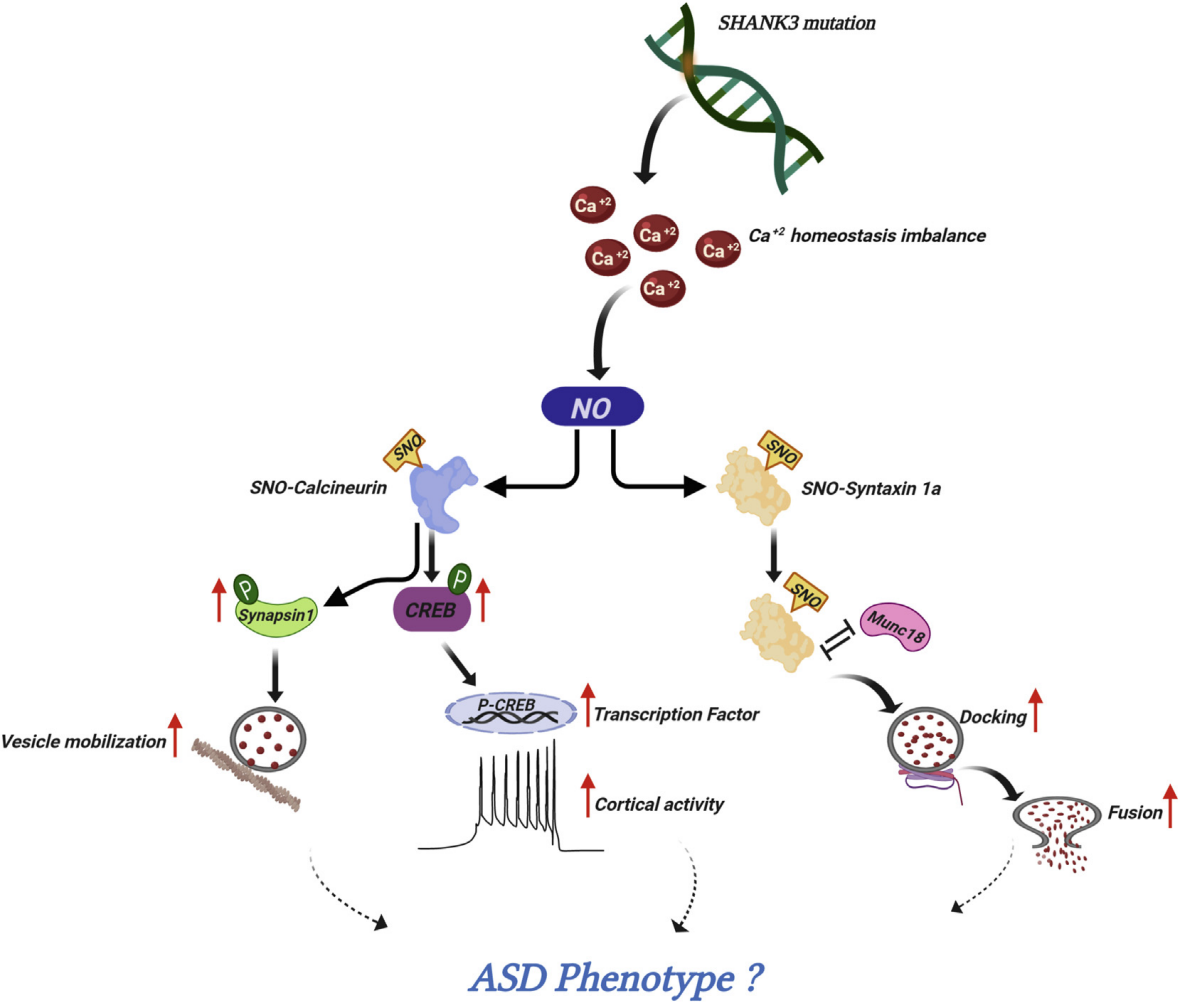
NO signalling in autism spectrum disorder (ASD). Schematic representation of NO involvement in ASD. Mutation in *SHANK3* gene may cause imbalance in Ca^+2^ homeostasis. Ca^+2^ is responsible for intracellular NO production which leads to S-nitrosylation of many proteins. S-nitrosylation of calcineurin inhibited its phosphatase activity which leads to increased levels of phosphorylated (P) synapsin-1 and CREB. P-synapsin-1 increases vesicle mobilization and P-CREB increases the recruitment of transcriptional co-activators and cortical activity. S-nitrosylation of syntaxin1a, inhibited its binding with Munc-18 which ultimately leads to increased vesicle docking and fusion.

**Fig. 3:**
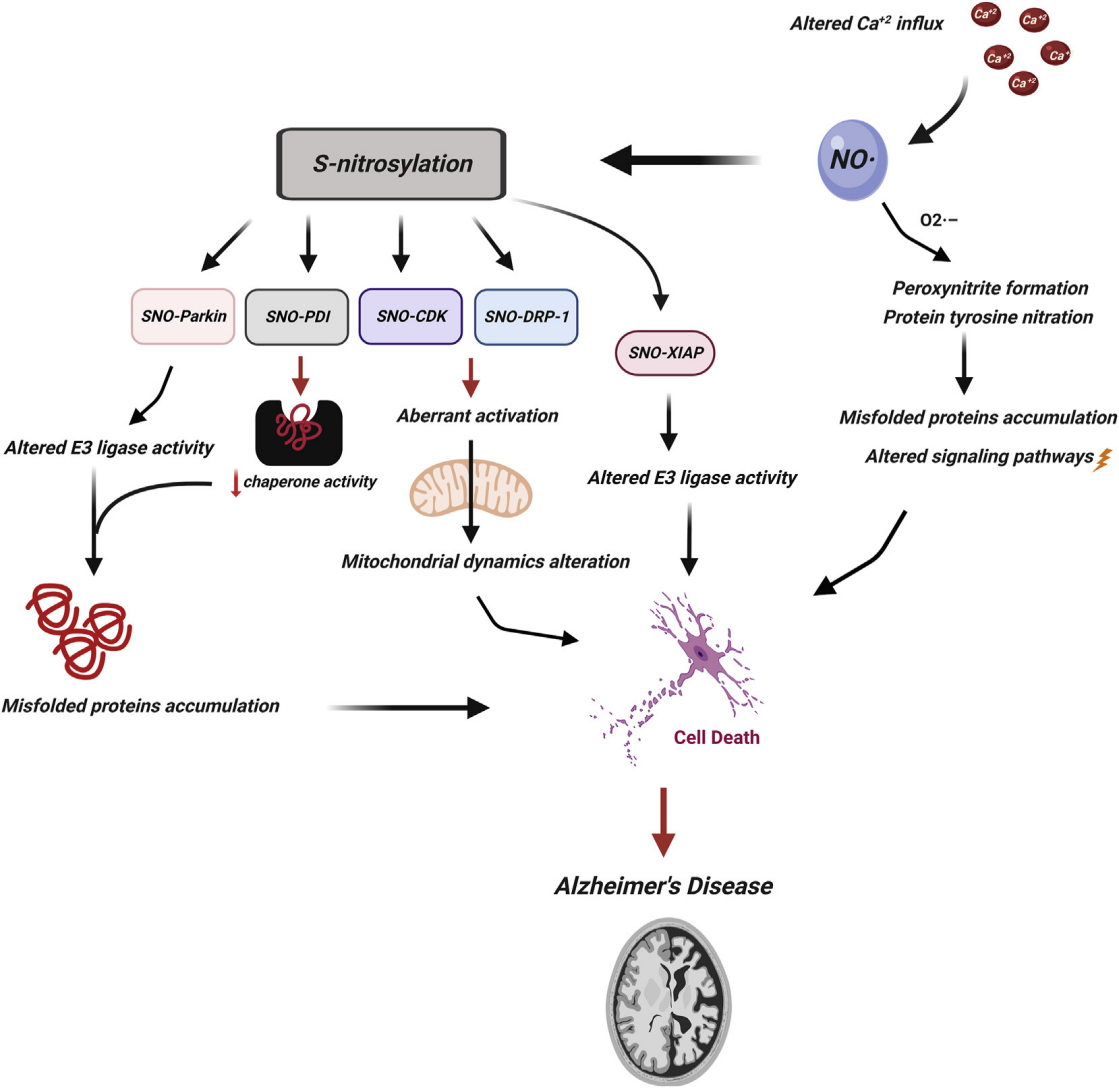
NO signalling in Alzheimer's disease (AD). Schematic representation of the involvement of NO in AD progression. Altered Ca^+2^ influx leads into aberrant NO production in cells, which S-nitrosylates many proteins and increases nitrosative stress, peroxynitrite formation, protein tyrosine nitration, which alters the signalling pathways and lead into cell death in AD. SNO of parkin and XIAP alter their E3 ubiquitin ligase activity. SNO of PDI disrupts its chaperone activity which enhances the accumulation of misfolded proteins in cells. SNO of Cdk and DRP-1 alters the mitochondrial dynamics.

**Fig. 4:**
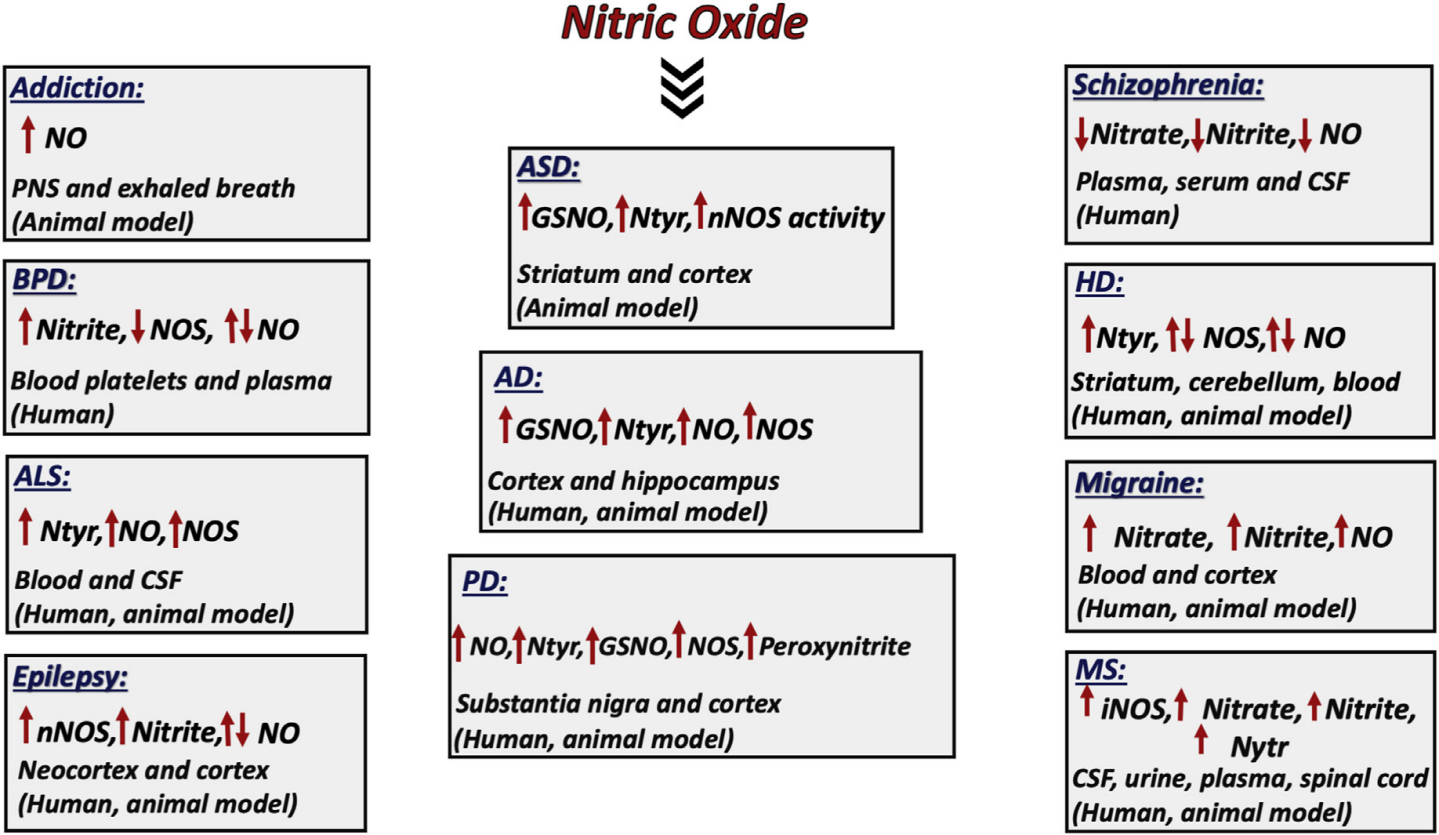
The involvement of NO in brain disorders. Alterations in NO and other NO-related molecular changes in the different brain disorders are presented. Abbreviations: NO: nitric oxide; Ntyr: nitrotyrosine; GSNO: S-Nitrosoglutathione; nNOS: neuronal nitric oxide synthase; iNOS: inducible nitric oxide synthase.

In this review, we summarize the role of NO and SNO in the progression of neurodevelopmental disorders, paying special attention to autism spectrum disorder (ASD). We discuss the involvement of SNO in the pathogenesis of ASD (See Fig. 2), which we have recently discovered in our studies [19]. We have summarize the involvement of NO and SNO signalling in Alzheimer's disease (AD) (Fig. 3). We also summarize the involvement of NO and SNO signalling in a number of other brain disorders including psychiatric, neurodevelopment, and neurodegenerative ones. Alterations in NO and other NO-related molecular changes in the different brain disorders are described in detailes (Fig. 4). Further, we list the key SNO-proteins involved in different brain disorders (Table 1). Table 2 summarizes different pharmacological agents used for therapies by manipulating NO. Finally, we provide mechanistic insight into the contribution of NO in diverse brain disorders and suggest potential and promising therapeutic targets for treatment.

**Table 1:** List of S-nitrosylated proteins involved in diverse brain disorders.

Protein	Disease		Sites of SNO modification		Experimental models	Suggested molecular/biological consequences of S-nitrosylation	Reference
Calcineurin	ASD		–		Mice	Inhibition of the phosphatase activity	[19]
Syntaxin 1a	ASD		–		Mice	Enhanced binding with SNARE complex	[19]
mGluR7	ASD		–		Mice	Increase of Ca^2+^ influx in presynaptic neurons	[19]
RNF213	AD		–		Mice	Inhibition of the ligase activity	[122]
Drp1	AD, HD		Cys-644		Human, mice, cell lines	Increase the rate of mitochondrial fission	[130,178]
Cdk-5	AD		Cys-83 and Cys-157		Human, mice, cell lines	Hyperactivation of the kinase activity	[125,126]
PSD 95	AD		Cys-3 and Cys-5		Animal, cell lines	Blocks translocation of the protein	[205]
IDE	AD		Cys-110 and Cys 819		Cell lines	Inhibition of the metalloprotease activity	[9,18,206]
ApoE	AD		Cys-112		Human, cell lines	Reduction of affinity to LDL receptors	[136,207]
MEF2	AD		Cys-39		Human, mice, cell lines	Reduction of the binding affinity to DNA	[41]
Aldolase C, fructose bisphosphate	AD		–		Human	Reduction of the metalloprotease activity	[137]
MAP1B	AD		Cys-2457		Mice, cell lines	Enhancement of the binding affinity with microtubules	[36,208]
Carbonic anhydrase-II (CAH-II)	AD		–		Human	Reduction of the enzymatic activity leading to protein accumulation	[209]
Caspases	AD		Cys-163		Human cell lines	Decline in protease activity	[9,210]
GSK3β	AD	Cys-76, cys-199, cys-317		Mice, cell lines		Inhibition of the kinase activity and increased translocation into nucleus	[119]
XIAP	PD, AD, HD	–		Human, mice, cell lines		Inhibition of the anti-apoptotic function	[150]
GAPDH	AD, ALS, cerebral ischemia, PD	Cys-150, cys-152		Human, mice, cell line		Enhancement of the binding with Siah complex and activation of p300/CBP resulting in the increased neuronal death	[33,183,184]
Parkin	PD	–		Human, mice, cell lines		Autoubiquitination and degradation.	[147,152]
DJ-1	PD	Cys-46, cys-53, cys106		Human, cell lines		Reduction of the antioxidant activity	[148,211]
PTEN	PD, AD	Cys-83		Human, cell lines		Reduction of the phosphatase activity	[148]
PDI	PD, ALS	–		Human, mice, cell line		Inhibition of the dithiol isomerase activity	[133,149]
Prx2	PD	Cys-51, cys-172		Human, cell lines		Inhibition of the antioxidant activity	[151]
Huntingtin	HD	–		Mice, cell lines		Protein aggregate formation leading to cell death	[174]
NMDAR	AD, Prion disease	Cys −744, cys-798		Animal		Inhibition of the receptor activity and NO production	[9,18,212]
MMP9	Cerebral ischemia	–		Animal, cell lines		Reduction of the metalloprotease activity	[213]
PLP	MS	–		Animal		Conformational and functional alteration	[195,204]

**Table 2:** Summarization of different pharmacological agents used for therapies by manipulating NO.

Disease name	Pharmacological agents	Protective versus detrimental effects	References
Schizophrenia	Sodium nitroprusside	Improvement in attention, cognitive and working memory	[68,69]
Bipolar disorder	Lithium	Increased NO level in plasma and improved symptoms.	[76]
Migraine	1 Sodium nitroprusside 2 Isosorbide dinitrite 3 Sildenafil 4 Mepyramine	1-Initiated headache and migraine 2 Initiated migraine 3 Increased migraine pain 4 Reduced headache	[[87], [88], [89],[94], [95], [96]]
Epilepsy	1-Methylene blue 2 l-arginine 3 l-NAME	1-Increased symptoms of epilepsy 2 Reduced symptoms of epilepsy 3 Induced seizures	[99,100,102,103]
Addiction	1-l-NAME 2 L-NNA 3-Tempol	1 Reduced morphine abstinence syndrome 2-Reduced opioid withdrawal syndrome 3-Abolished cocaine psychomotor sensitization	[[108], [109], [110],^112^]
AD	L-NNA	Reduced apoptosis	[126]
PD	1-7 -nitroindazole 2 Pargyline 3 l-NAME 4 GW274150	1-Protection from cell death 2 Protection from cell death 3 Protection from cell death 4 Reduced microglial activation, cytokine and NO production	[[164], [165], [166], [167],^170^]
ALS	1 Deprenyl 2 Diethyl NONOate 3 L-NNA	1 Reduced apoptosis 2 Reduced cell death 3 Reduced aggregates formation	[183,185,191]

## 2 Role of nitric oxide in neuronal development

NO plays major roles in neurogenesis and neurodevelopment [20]. Importantly, NO regulates the activity of the brain-derived growth factor (BDNF) [21]. BDNF promotes SNO of many nuclear proteins, including those related to the cAMP response element-binding protein (CREB), a cellular transcription factor that is involved in regulation of neuronal and dendritic development [22]. Sen and Snyder [23] have shown that BDNF, along with other nerve-growth factors, activate nNOS leading to S-nitrosylation of glyceraldehyde 3-phosphate dehydrogenase (GAPDH)/seven in absentia (Siah) homolog complex (SNO-GAPDH-Siah). SNO of GAPDH at Cys-150 promotes its association with Siah, which leads to formation of SNO-GAPDH-Siah complex. Further, SNO-GAPDH-Siah complex translocate into the nucleus [24], initiating the ubiquitination and degradation of the histone-methyltransferase enzyme, suppressor of variegation 3–9 homolog 1 (SUV39H1) protein. SUV39H1 is the principal enzyme responsible for trimethylation of histone H3 at Lys-9, a molecular marker associated with transcriptional silencing. Therefore, inhibition of the trimethylation of histone H3 via SUV39H1 degradation enhances the binding and activation of CREB [23]. In conjunction with BDNF-induced SNO, CREB binds to the cAMP response element of the promoters of its target genes upon phosphorylation at Ser-133 in the KID domain [25] by different receptor-activated protein kinases, such as protein kinase A (PKA), calmodulin-dependent protein kinase (CaMK), mitogen-activated protein kinases (MAPK), and other kinases. These kinases are activated by Ca^2+^ influx triggered upon depolarization [26,27]. The phosphorylation of Ser-133 leads to a 10- to 20-fold increase in CREB's transcriptional activity [28]. Interestingly that both NO signalling, which produces cGMP, and cAMP signalling could regulate the phosphorylation of CREB (see Fig. 1). cAMP activates CREB through the canonical cAMP/PKA pathway and the exchange protein directly activated by cAMP (Epac) pathway. Epac activates extracellular signal-regulated kinase 1/2 (ERK1/2) signalling, which subsequently leads to Ser-133 phosphorylation of CREB [29]. Meanwhile, cGMP activates the downstream protein cGMP-dependent protein kinase G (PKG), which also phosphorylates the transcription factor CREB at Ser-133 [29]. This dual phosphorylation by cAMP/PKA/Epac and cGMP/PKG pathways may amplify the CREB activity [29]. Once CREB is activated and CREB-binding protein is recruited, transcription is initiated [30].

Further, under normal NO concentration, SNO-GAPDH-Siah translocation is negatively regulated by GAPDH's competitor of Siah protein enhancer life (GOSPEL) protein. SNO of GOSPEL at Cys-47 enhances its ability to bind to GAPDH, which terminates the translocation of SNO-GAPDH-Siah complex. SNO-GAPDH-GOSPEL remains in the cytosol [31]. In contrast, nuclear translocation of SNO-GAPDH promotes transnitrosylation of many proteins, such as histone deacytylase 2 (HDAC2), DNA-activated protein kinase, sirtulin −1, and others [32,33]. BDNF-induced transnitrosylation reaction between SNO-GAPDH and HDAC2 results in SNO of HDAC2 at Cys-262 and Cys-274 [34]. Normally, HDAC2 remains attached to CREB target gene promoters. However, SNO of HDAC2 promotes its dissociation and rapid acetylation of histone H3 and H4, resulting in association of CREB with its target genes. Consequently, BDNF-mediated SNO of HDAC2 plays a role in dendritic development [32,34].

Rearrangement of actin and myosin in cytoskeletons is necessary for axonal growth, axonal guidance, axonal modification and brain development [20] and these processes are also mediated by NO [35]. Furthermore, SNO of microtubule-associated protein B1 (MAPB1) leads to a modification of axon retraction [36]. MAPB1 contains a heavy chain (HC) and a light chain (LC1) domains. LC1 domain can be S-nitrosylated at Cys-2657 of MAPB1, which increases the binding capacity of HC/LC1 MAPB1 complex with microtubules. This complex inhibits the dynein leading to inhibition of axonal extension and increases of axonal retraction [36].

Previous work has shown the involvement of NO in neurogenesis [[37], [38], [39]]. nNOS knockout rats or inhibition of nNOS by pharmacological agents negatively regulate neurogenesis [[37], [38], [39]]. Thus, SNO of myocyte enhancer factor 2 (MEF2), a transcription factor involved in neurogenesis at Cys-39 reduces its binding affinity to DNA and ultimately inhibits its transcriptional activity [40]. SNO-MEF2 also reduces the expression of nuclear receptor tailess (TLX) [41], which is a regulator of adult neurogenesis and is responsible for learning and memory [42]. Lipton and co-workers have found high level of SNO-MEF2 in Alzheimer's disease (AD), leading to neurodegeneration. These changes were present in the post-mortem brains and mutant transgenic mice [41]. Thus, SNO-MEF2 inhibits neurogenesis and neuronal differentiation in brain [41]. Studies on nNOS knockout mice showed abnormal dendritic branching [43] and reduction in neurogenesis [37]. All these reports imply that NO plays an important role in neurodevelopment. Thus, dysregulation of the NO signalling can bring about a variety of neurodevelopmental diseases. Below, we discuss the role of NO in different brain disorders.

## 3 Autism spectrum disorder (ASD)

ASD is a neurodevelopmental disorder associated with impaired communication, impaired social skills and repetitive behaviour [44]. ASD is caused by genetic mutations, as well as environmental and non-genetic factors [45]. According to the world health organization (WHO), 1–1.5% of children suffer from ASD globally [46,47]. Currently, there is no treatment for ASD and symptomatic features are reduced by different psychiatric medications.

SHANK3 mutation is one of the most promising ASD-associated mutations [48]. Several reports on *Shank3* KO mouse models showed defects in biochemical, electrophysiological and cellular pathways [[49], [50], [51]]. As per our knowledge, Amal et al. was the first to report the involvement of NO in the development of ASD [19]. Amal et al. has hypothesized that *Shank3* mutation leads to an increase of Ca^2+^ influx that in turn activates nNOS activity leading to the dramatic NO formation and NO-related molecular changes, including S-nitrosoglutathione (GSNO), 3-nitrotyrosine (Ntyr), and SNO [19]. SNO targets a wide range of prominent intracellular proteins leading to alteration in signalling pathways, which may converge onto synaptic, neuronal and behavioral deficits. The work has reported that in *Shank3* mutated mice [InsG3680 (+/+)], the SNO-proteome is reprogrammed and dysregulation of proteins by S-nitrosylation and de-nitrosylation occurs [19]. System biology analysis of both wild type (WT) and *Shank3* KO mice revealed 9-fold change in SNO level of proteins involved in the synaptic vesicle cycle (Syntaxin1a (Stx1a), synaptotagmin 1, and N-ethylmaleimide sensitive fusion protein (Nsf)) in cortex of KO mice but not in WT mouse brain. Gene ontology (GO) and KEGG analysis of 6-week-old KO mice showed enrichment of many proteins that involved in neurodevelopment and ASD. Further, systems biology analysis showed the enriched SNO proteins involved in synaptic vesicle cycle and oxidative phosphorylation in *Shank3* KO mice. These results convincingly show an association between S*hank3* mutation and NO [19]. Further, this work showed that protein-protein interaction analysis in the cortex of KO mice showed a network of S-nitrosylated proteins functionally involved in synaptic vesicle cycle, neurotransmission (protein phosphatase catalystic subunit alpha-Ppp3ca, syntaxin 1a, vesicle associated membrane protein 3 and others) and in glutamatargic pathway (glutamate dehydrogenase 1, mGluR, G protein subunit alpha O1 Gnao-1 and others) [19]. Analyzing the shared SNOed proteins in the cortex of KO mice of both 6-week-old and 4-month-old mice showed an evidence of enriched processes known to be affected in ASD, such as synaptic vesicle cycle. The interactome analysis of the shared proteins in the cortex of KO mice showed protein clusters that function in the synaptic vesicle cycle (syntaxin 1a, Ppp3ca, Nsf and Dnm1) and glutamate regulation (glutamic-oxaloacetic transaminase-Got1, Got2, Gnao-1). This work showed an increase of 3-nitrotyrosine level in different cortical regions. Level of GSNO was found to be increased in the cortex of both KO groups as compared with WT groups. The study also showed that calcineurin was SNOed in the cortex which inhibited its phosphatase activity (see Table 1 and Fig. 2). Inhibition of calcineurin activity increased the levels of *p*-Synapsin-1 and *p*-CREB protein [19], (see Fig. 2). Synapsin-1 is involved in regulation of vesicle exocytosis and its phosphorylation increases exocytosis of vesicles [52]. Increase in phosphorylated level of Synapsin-1 in the cortex of the mutant mice may indicate that SNO of calcineurin is responsible for increased vesicle mobilization. The study found a significant increase in *p*-CREB levels, which is another substrate of calcineurin [19]. Increased level of *p*-CREB has also been reported in another model of ASD [53]. Syntaxin1a, which enhances the formation of the SNARE complex, was SNOed in *Shank3* KO mice [19]. SNO of this protein enhances the formation of the SNARE complex leading to increase of synaptic vesicle docking and fusion [54], (see Fig. 2). SNO of metabotropic glutamate receptor 7 (mGluR7) was found in the cortex of the mutant mice. The study suggested that SNO of mGlur7 may increase the influx of Ca^2+^ in presynaptic neurons, which in turn increases vesicle fusion [19]. Taken together, the study implies that NO is an important factor in ASD. The insights obtained from the *SHANK3* mutation study may likely be applicable to a broader group of patients with genetically diverse but mechanistically related etiology, thus it may imply NO as an important pathological factor in ASD.

## 4 Schizophrenia

Schizophrenia is a severe and chronic mental disorder, that affects person's thinking, feeling, and behaviour. It accounts for approximately 1% of the total world population [55]. The cause of schizophrenia is unknown and it is considered as a multifactorial disorder [56]. Symptoms of schizophrenia can be divided into three different categories, such as positive symptoms, negative symptoms and cognitive disturbances [56,57]. Hallucinations, catatonic behaviour, delusion and disturbed thought procession are considered as positive symptoms. Avolition, anhedonia and social withdrawal represent negative symptoms [55]. Studies suggested that NO may play a role in the development of schizophrenia [58,59]. Researchers hypothesized that impairments of dopaminergic and cholinergic pathways may be involved in the development of schizophrenia through the involvement of NMDA receptors-dependent NO signalling [[60], [61], [62]]. Polymorphism of nNOS gene is found to be a critical risk factor for the development of schizophrenia [63]. Also, the levels of NO metabolites, such as nitrite and nitrate, were found to be reduced in plasma, serum and cerebrospinal fluid (CSF) of schizophrenic patients (see Fig. 4) [[64], [65], [66]]. Previous work showed that the genetic ablation of nNOS results in cognitive deficit in mice, emphasizing the role of NO in learning and memory [67]. Schizophrenic patients that were treated with NO donor nitroprusside, showed improvement of symptoms (attention, cognitive and working memory) (see Table 2) [68,69]. All these studies suggested that NO deficiency plays a role in the pathology of schizophrenia.

## 5 Bipolar disorder (BPD)

BPD is a chronic mental illness also referred to as manic depressive illness [70]. Accurate and early diagnosis of BPD is difficult and more than 1% of individuals suffer from this kind of neuropathology globally [71]. On the basis of severity and duration of the manic and depressive episodes, the Diagnostic and Statistical Manual of Mental Disorders, Fifth Edition (DSM-5) [72] divide BPD into four categories, bipolar I disorder, bipolar II disorder, cyclothymia and residual category [70]. It has been revealed in some studies that defects in dopaminergic and serotonergic pathways are responsible for the development of BPD [70,73]. The role of NO signalling in BPD has also been reported [74]. NOS activity was reduced in blood platelets of patients suffering from BPD compared to healthy individuals [75]. Another study has shown that lithium treatment increases the level of NO in plasma of patients suffering from this disease, indicating a role of lithium in regulation of NO signalling in subjects with this pathology during depressive episodes (see Table 2) [76]. In this study, plasma NO level in bipolar depression was not different from healthy controls. Other works revealed that NO and nitrite level in plasma of bipolar patients was higher than in healthy controls (see Fig. 4) [77]. A meta-analysis carried out by Andreazza et al. has confirmed an increased activity of the NO signalling in patients with BPD [78]. The controversy of these data can be explained by the differences in the stage of the disease and medications used [76]. Nevertheless, the accumulated data can show the involvement of NO in BPD, although its mechanistic role needs further investigation.

## 6 Migraine

Migraine is a reversible and chronic neurological disorder with severe or moderate headache. The major symptoms associated with migraine are cutaneous allodynia, phonophobia, photophobia, dizziness, vertigo and different gastrointestinal problems [79]. According to WHO, migraine is the second most common disabling neurological disorder and the third most common medical condition of the world, in which 33% of women and 13% of men are suffering from it [79,80]. The role of NO in migraine was reported many years ago. Blood sample analysis of migraine patients revealed an increased level of cGMP, nitrite, neurokine A and calcitonin gene-related peptide (CGRP), which indicates the association of NO with migraine [81,82]. A variety of factors affect the formation and release of NO, such as bradykinin, NMDA, 5-hydroxytryptamine (5-HT_2B/C_) receptors, substance P, histamine, acetylcholine, and others [[83], [84], [85]], which indicates its involvement in central pain sensation. Altogether, these data indicate the involvement of NO in the mechanisms of migraine and headache [82,86]. Glyceryl trinitrite (GTN), or sodium nitroprusside, was found to initiate headache and migraine symptoms in workers at explosive industry. This was the first direct evidence of the role of NO in migraine [87]. Further studies of NO donor molecules, such as GTN and isosorbide dinitrate, on migraineurs (people who are suffering from migraine) and non-migraineurs showed that migraineurs are more sensitive to the NO donor compounds than non-migraineurs [88,89]. Nitrate and nitrite concentration in blood was higher in migraineurs compared to control patients (see Fig. 4). These results indicate that NO is involved in initiation and maintenance of pain in migraine [90]. In animal models, headache feature was not investigated, but biochemical analysis has been done. Treatment with GTN in rats resulted in the increase of nNOS in trigeminal nerve ending, NO in cortical regions, and c-fos expression in trigeminal nucleus caudalis [82,91]. Along with this, reduced superoxide dismutase (SOD) expression and increased cortical blood flow were also found in animal models after GTN injections [92,93]. Treatment with cGMP-hydrolyzing phosphodiesterase 5 inhibitor, Sildenafil, led to increased migraine pain in migraineurs compared to controls [94]. Histamine is reported to induce headache in migraineurs and use of a histamine antagonist, Mepyramine, reverted this effect (see Table 2) [95,96]. However, when the NOS-inhibitor L-NG-methylarginine hydrochloride (was administered to histamine-induced migraine patients, no significant changes were found [97]. Nevertheless, in general, the published data indicate that NO signalling may play a role in the development of migraine.

## 7 Epilepsy

Epilepsy is the most common neurological disorder that affects approximately 50 million people worldwide [98]. It is characterized mainly by recurrent seizures accompanied by loss of consciousness [98]. Here we discuss the involvement of NO in epilepsy. NO regulates excitatory and inhibitory neurotransmission in both physiological and pathological conditions [99]. There is some controversy over the role of NO signalling in the development of epilepsy. Previous reports indicated that NO plays a role of anticonvulsant while other showed that it leads to pro-convulsant effect [99]. Thus, rats with epilepsy induced by NMDA injection were treated with methylene blue, known as a nNOS inhibitor. The methylene blue treatment increased the symptoms of epilepsy [100]. It is worth mentioning that methylene blue appears to be a guanylate cyclase inhibitor that does not interfere with NOS [101]. This casts doubt on the conclusion of the above-mentioned [100] study. Nevertheless, when a precursor of NO l-arginine was given, it reduced the symptoms of epilepsy [99,102]. Also, in DL, homocysteine-thoiolactone (H)-induced seizure model, l-arginine provided protection and the NOS inhibitor N(G)-nitro-l-Arginine methyl ester (l-NAME) treatment potentiated the incidence of seizure (see Table 2) [103]. The researchers concluded that NO is responsible for protection against epilepsy. However, in another study, increased level of NMDA subunit NR2B receptor in epileptic dysplastic human neocortex indicated the involvement of NO [104] and overexpression of nNOS [105] in the pathogenesis of epilepsy was found. In Pentylenetetrazol (PTZ)-induced epilepsy in rats, overexpression of NO and lipid peroxidation was reported in the brain and antioxidant treatment normalised the level of both (see Fig. 4) [106].

## 8 Addiction

Addiction is a brain disorder distinguished by compulsive engagement in rewarding stimuli. To test the involvement of NO in addiction, morphine addict rats were treated with NOS inhibitors, l-NAME and nitro-l-arginine (L-NNA) and tested the drug withdrawal symptoms. The rats showed reduced morphine withdrawal symptoms and when treated with NO donor compound, isosorbide nitrate, relapse of withdrawal symptoms were detected [108,109]. Immunohistochemical studies in morphine-dependent mice showed increased number of nNOS positive cells in olfactory bulb, cerebellum, medulla oblongata, and locus coeruleus and reduction in the hypothalamus. When treated with an opioid receptor antagonist naloxone, increased nNOS immunoreactivity was found in hypothalamus [110]. These results can explain the role of NO in opioid dependence and withdrawal symptoms [110]. Cocaine is also responsible for induction of nNOS activity in hippocampus of rat brain [111] and tempol treatment, an antioxidant agent, abolished cocaine psychomotor sensitization and condition reward via reduction in oxidative stress [112]. Studies on alcohol addiction showed that ethanol reduces the NO synthesis in peripheral system and also the level of NO in exhaled breath (see Fig. 4) [113]. When l-NAME (an inhibitor of NO) was given to rats, it led to increase in ethanol-induced narcosis; and when isosobarbide dinitrate (an NO donor) was applied, the effect of necrosis was reduced in rats [114]. Further, treatment with 7-nitroindazole(7-NI) (an inhibitor of nNOS) enhances hypnotic effect of ethanol in animals (see Table 2) [115]. Following the mentioned evidence, we suggest that NO signalling plays an important role in addiction.

## 9 Alzheimer's disease (AD)

Alzheimer's disease (AD) is the most common chronic neurodegenerative disorder [116]. Aging is the main causative factor for the development of AD and memory loss [116]. Cognitive deficits and language impairment are major symptoms of AD [117,118]. Senile plaques and neurofibrillary tangles are the main pathological hallmarks of AD [117]. Tannenbaum and co-workers have tested the involvement of SNO in AD using SNO trapping by TriAryl Phosphine (SNOTRAP) method in conjunction with mass spectrometry [119]. They used the CK-p25-inducible mouse model of AD, and a total of 251 SNO-proteins were detected in the AD model. Among them, 135 SNO proteins were found exclusively for early neurodegeneration in cortex [119]. These proteins found are known to be associated with metabolism, synaptic function and AD progression [119]. According to the GO analysis of CK-p25 mouse model of AD, increased number of SNO proteins were found in cortex and hippocampus compared to control mice. Increased level of amyloid β protein, DNA damage, neuroinflammation and behavioral abnormalities were observed in CK-p25 mice. An increase in GSNO was found in hippocampus and cortex but not in cerebellum of 2-week-old CK-p25 mice. SNO level of PSD95 was also detected in these mice. SNO proteins associated with AD, such as glutamate ionotrophic receptor NMDA 2B (Grin2b), microtubule associated protein (MAPT), glycogen synthase kinase 3β(Gsk3b), lipoprotein receptor-related (LRP), NADH, ubiquinone oxidoreductase core subunit S1 (Ndufs1), cytochrome *c* oxidase subunit 6B1(Cox6b1) and GAPDH were detected in cortex of Ck-p25 mice brain. Elevated phosphorylation of GSK3β and tau (Mapt) was found in the transgenic mice. It has been shown that these proteins are involved in neuronal cell death in AD [120]. PKCε and PKCγ, isoforms of PKC were found to be SNOed in CK-p25 mice brain. Lipoprotein receptor-related protein (LRP) gene is associated with progression of AD [119,121]. Researchers suggested that SNO-LRP and SNO-PKC in CK-p25 mice disturbed amyloid β processing and clearance, which further lead to amyloid plaque deposition [119]. Amal et al. used P301S mouse model of tauopathy to test the involvement of SNO in the pathology [122]. This work revealed reprogramming of the S-nitroso-proteome in the mutant compared to the control group in both cortex and hippocampus of 2-month-old mice. Increased level of 3-nirotyrosine in the CA1 and entorhinal cortex regions of P301S mice was found. This indicates nitrosative and oxidative stress in the brain of the mutant. This study revealed the role of the noncanonical Wnt/Ca^2+^ (NC-WCa) signalling in the cortex of P301S mice and found an elevated level of phosphorylated CaMKII. Ring Finger Protein 213 (RNF213), an E3 ubiquitin ligase, was S-nitrosylated, and led to an increase in the level of nuclear factor of activated T-cells 1 (NFAT-1) and FILAMIN-A, which resulted in the potentiation of the NC-WCa signalling [122].

Cyclin dependent kinase (Cdk5) is a serine/threonine kinase enzyme which has diverse functions in the development of brain, synaptic plasticity, regulation of neuronal migration and differentiation [123]. Dysregulation/dysfunction or modification of Cdk5 leads to the development of many neurological disorders [123]. In neuronal cells, Cdk5 scaffolds with the nNOS/PSD95/NMDAR complex which are embedded in the membrane. Cdk5 is S-nitrosylated by interacting with nNOS complex under pathological conditions [124,125]. Post-mortem studies revealed the presence of high level of SNO-Cdk5 protein in the AD brain compared to the normal brain [126]. S-nitrosylation of Cdk5 at Cys-83 and Cys-157 residues activate the function of Cdk5 (see Table 1) [124,125]. Activation of Cdk5 leads to phosphorylation of its substrate ataxia telangiectasia mutated kinase (ATM), which is a proapoptotic protein kinase. In cultured cortical neurons, SNO-Cdk5 is found to enhance the amyloid β (Aβ)-induced synaptic degeneration [126]. Cdk5 mutant protein or treatment with NOS inhibitor, N-nitro-l-arginine (NNA), in the cellular system provided protection against apoptosis (see Table 2) [126]. In summary, aberrant SNO of Cdk5 is responsible for Aβ-induced synaptic degeneration (see Fig. 3) [126]. Importantly, NMDAR plays a role in Aβ-induced synaptic loss [127,128]. Cdk5 activates NMDAR via phosphorylation and *p*-NMDAR activates nNOS in ischemic insult [129]. The resulting SNO-Cdk5 transnitrosylates Drp-1 protein, a GTPase protein that regulates mitochondrial dynamics such as mitochondrial fission and fusion in cells, with detrimental effects. For example, treatment of the primary cortical neurons with Aβ led to SNO of Drp-1 at Cys-644 residue, which resulted in enhanced mitochondrial fission, impairment of energy homeostasis and dendritic spine loss (see Table 1) [130]. A mutant of Drp-1 (C644A) protected the cells from Aβ-induced synaptic loss [130]. Post mortem studies have confirmed the presence of SNO-Drp-1 in the brain of AD patients and peripheral blood lymphocytes but not in controls [131,132]. The researchers concluded that S-nitrosylation of Drp-1 plays a major role in AD progression (see Fig. 3) [131,132].

Protein disulphide isomerase (PDI) is a chaperon protein, located in endoplasmic reticulum (ER) and plays a major role in protein processing and folding. SNO-PDI was found in post-mortem AD brain [133]. PDI was S-nitrosylated in its active site and inhibited its activity, which led to accumulation of unfolded proteins (see Fig. 3) [133]. Furthermore, a zinc metalloprotease insulin degrading enzyme (IDE) was found to be S-nitrosylated at multiple sites (Cys819, Cys789, Cys966, Cys178) in the presence of GSNO (see Table 1). S-nitrosylation of IDE was found to be involved in the pathogenesis of AD [134]. Mutation in Apo lipoprotein E (ApoE) was reported in late onset of AD [135]. ApoE appears to be S-nitrosylated in patients with AD, and recent studies have reported hippocampal SNOs of ApoE2 and ApoE3. These studies suggest that SNO of ApoE proteins may play a role in AD development by inhibiting lipid homeostasis (see Table 1) [136]. SNO-GAPDH has also found to be S-nitrosylated in various brain regions including cortex, substantia nigra, and hippocampus in postmortem AD brain compared to control brains [137], which suggested the involvement of SNO-GAPDH in AD [137].

Thus, SNO plays a crucial role in the pathogenesis of AD, affecting the activity of numerous proteins. We believe that unravelling the SNO-related signalling pathways will pave the way to new effective therapies against the development of AD.

## 10 Parkinson's disease (PD)

Parkinson's disease (PD) is the second most common disease after AD in elderly people [138]. It is characterized by four major cardinal features: bradykinesia, rigidity, postural instability and resting tremor [139]. In PD, NO plays both a neuroprotective and neuro-destructive role and was reported in rodent models and human cases of PD [9,140,141]. S-nitrosylation of many proteins was found to be involved in PD progression [9]. Biochemical analysis of postmortem PD brains revealed increase in oxidative/nitrosative stress, which plays a major role in PD pathogenesis [142]. NO forms peroxynitrite in the presence of hydrogen peroxide [143]. Production of peroxynitrite leads to the modification of α-synuclein protein by di-tyrosine synthesis, which further stabilizes the filamentous structure of α-synuclein resulting in aggregate formation [144]. Analysis of postmortem PD brains in substantia nigra and cortical regions showed increased level of nitrated α-synuclein [145]. Nitrated α-synuclein was also found in substantia nigra of 1-methyl-4-phenyl-1,2,3,6-tetrahydropyridine (MPTP) mice model of PD [146]. S-nitrosylation of different proteins, such parkin [147], DJ-1 [148], PDI [133,149], X-linked inhibitor of apoptosis (XIAP) [150] and Prx2 [151] were found to be associated with PD (see Table 1). Previous works have shown that nitrosative stress led to S-nitrosylation of parkin, causing inhibition of its ubiquitination activity, which lead to the formation of Lewy bodies and PD progression. These changes were found both in rodents and in human postmortem PD brain [147,152]. Further, human studies confirmed the SNO modification of parkin in PD pathogenesis [147,152,153] as well as in different mouse models of PD [140,152].

PDI is a chaperone protein residing inside the endoplasmic reticulum and contributing to protein folding and disulfide formation by its isomerase activity [154]. Researchers found that S-nitrosylation of PDI inhibits its activity [133]. They have showed that toxicant rotenone increases the S-nitrosylation of PDI in SH-SY-5Y cell lines [133]. Further, SNO-PDI was also found in the brain of postmortem PD patients, which may emphasize its involvement in the pathology [133,155].

XIAP is an E3 ubiquitin ligase protein which participates in ubiquitination and degradation of caspase 3,7,9 and promotes cell survival [156]. High level of NO leads to SNO modification of XIAP and inhibition of its ubiquitination activity. In rotenone-treated HEK-293 and N2a cell line models of PD, S-nitrosylation of -XIAP is well reported (see Table 1) [150]. Human studies on PD patient's postmortem brain also confirm the SNO modification of XIAP [150,157].

Prx2 has antioxidant properties since it catalyzes the removal of peroxides from cells, and its level is upregulated when oxidative stress is increased in the cells [158]. S-nitrosylation of Prx2 at Cys-51 and Cys-172 inhibited its antioxidant activity [151], which leads to increase of oxidative stress. Importantly, increased level of SNO-Prx2 is reported in PD human postmortem samples and PD animal models (see Table 1) [151].

PD toxicant rodent models, such as MPTP, 6-hydroxydopamine (6-OHDA), lipopolysaccharides (LPS), manganese ethylene bis-dithiocarbamate (maneb) and 1,1′-dimethyl-4,4-bipyridinium dichloride (paraquat), found to have high expression levels of iNOS, nNOS and NO in the brain (see Fig. 4) [[159], [160], [161], [162]]. iNOS-KO mouse model showed resistance to MPTP-induced cell death [163]. Furthermore, NOS inhibitors, 7-nitroindazole and monoamine B oxidase (MAO-B) inhibitor pargyline, protected cell death of dopaminergic neurons in mouse model of PD induced by MPTP(see Table 2) [164]. Together, these studies conclude that NO is involved in MPTP-mediated neurodegeneration. 6-OHDA, is another toxicant used to induce PD in rat, which its intoxication leads to the increased level of iNOS and NO in the rat brain. Pretreatment of animals with NOS inhibitor l-NAME protects from dopaminergic cell death in substantia nigra pars compacta (SNPc) and from dopamine depletion in striatum [165,166]. Intranigral injection of 6-OHDA caused activation of microglial cells, cytokine production, generation of free radicals and increase of NO production, which ultimately led to the death of dopaminergic neurons in PD [160]. GW274150, an iNOS inhibitor, reduced microglial activation, cytokine, ROS and NO production, and provided neuroprotection against the 6-OHDA-induced rodent model of PD (see Table 2) [167]. Rotenone, used as a toxicant to induce PD in rodents, inhibited mitochondrial complex 1 and induced oxidative stress [168]. Furthermore, it reduced the level of glutathione, which further leads to the increase in nitrite levels and apoptosis [169]. Rotenone enhanced NOS enzymatic activity, increased 3-nitrotyrosine (3NT) level, reduced dopamine content and promoted the degeneration of dopaminergic cells [170]. Treatment with the NOS inhibitor 7-NI reduced NOS activity and 3-NT level and provided protection against rotenone-induced toxicity (see Table 2) [170]. Rotenone can induce nitrosative stress by disrupting the redox activity of PDI [171]. In rat striatal synaptosomes treated with methamphetamine, which is also used to produce a toxic model of PD, activated alpha(7) nicotinic receptors and increased the level of intrasynaptosomal calcium, NOS and PKC [172]. All this led to cell death of dopaminergic neurons in the PD rat model [172]. The above data unequivocally point to the involvement of NO and nitrosative stress in the pathogenesis of PD.

## 11 Huntington disease (HD)

Huntington disease (HD) is a dominantly inherited autosomal neurodegenerative disorder [173]. The main features of HD are motor dysfunction, cognitive impairment and psychiatric disturbances [174]. The main cause of this disease is expansion of CAG repeats in Huntingtin (HTT) protein on chromosome number 4 [173]. The expansion of CAG repeats in Mutant HTT (mHTT) protein results in the formation of long polyglutamine (polyQ) repeats making mHTT a toxic protein. This causes dysfunction of normal homeostasis and cell death of the neurons [175]. The disease starts from the striatum, and when it progresses, it reaches the cortex [173]. Although not enough information is available on the involvement of NO in HD, it is known that the key proteins involved in the progression of HD also become S-nitrosylated. In R6/1 mutant HD mouse, the level of expression and biochemical activity of nNOS was found to be reduced in the striatum and cerebellum (see Fig. 4). Along with this, behavioral abnormalities were also reported in R6/1 HD mice [176]. In another study on SK-N-SH human neuroblastoma cell lines, knockdown of nNOS appeared to be protective for the cells. This can be explained by the fact that inhibition of nNOS may induce autophagy [177]. Mitochondrial dysfunction is the major cause of the development of neurodegenerative disorders. In this context, SNO of dynamin-related protein (Drp-1), the activator of mitochondrial fission, was found to be increased in the human postmortem HD brain and striatum of the transgenic HD mouse model [178]. Further investigation reported that SNO-Drp-1 disrupts the mitochondrial dynamics eventually leading to cell death [179]. Another study has revealed that PTM of HTT protein in N-548 fragment is implicated in the expansion of polyQ repeats. Overexpression of NOS is responsible for SNO of normal HTT protein (see Table 1) [174]. Investigation of the human HD patients indicate that abnormal NO signalling in the peripheral blood tissues is also responsible for the disease progression [178].

## 12 Amyotrophic Lateral Sclerosis (ALS)

Amyotrophic Lateral Sclerosis (ALS) is an idiopathic neurodegenerative disorder [180]. It is also known as Motor Neuron Disease. This disease is mainly characterized with permanent impairment of lower and upper motor neurons [181]. ALS is sporadic in nature in 85–90% of cases but genetic factors (10–15%) also contribute in the development of this disease. ALS is more common in men than in women [182]. Until now, approximately 30 genes responsible for the development of ALS pathogenesis have been detected [181]. Toxic gain of function due to mutations in SOD1 is one of the major culprits in ALS [180]. Mutant SOD1 ^G93A^ is responsible for SNO of GAPDH, which enhances the nuclear translocation of GAPDH-Siah complex in NSC34 motor neurons, which further enhances apoptotic cell death. When deprenyl, a selective inhibitor of Type B monoamine oxidase, was added, it inhibited the SNO of GAPDH and provided protection against apoptosis (see Table 2). This study concludes that mutations of SOD induce SNO of GAPDH, which plays a role in the neuronal apoptosis in ALS model [183]. In contrast, it has been found that mutations of SOD1 increase its denytrosylation activity, and denytrosylation of many mitochondrial proteins can hamper the normal homeostasis in mitochondria, ultimately leading to cell death in ALS models, whilst addition of SNO-donor compounds to the mutant SOD1-containing cells prevents this course of events [184]. This study implies that denytrosylation is important for the cell survival. This conclusion was confirmed by another study, where lymphocyte cells of ALS patients were treated with the NO-releasing agent diethyl NONOate. In this work, the cell death ratio was found to be lower in the diethyl NONOate-treated lymphocytes of ALS patients compared to controls [185].

The role of PDI in the development of ALS is well established. The level of PDI is increased in spinal cord and CSF of ALS patients [186] and spinal cord of SOD1 ^G93A^ transgenic rat [187] and mice [188] models of ALS. SNO of PDI in the spinal cord of ALS patients and SOD1 ^G93A^ transgenic mice was found to be responsible for the disease progression. Pharmacological agents that mimic the active site of PDI provide protection to SOD1 ^G93A^ transgenic mice [155]. Tyrosine nitration of PDI and ERp57 protein has also been reported in SOD1 ^G93A^ transgenic mice [189,190]. Treatment with NOS inhibitor, N-nitro-l-Arginine, inhibited both SNO of PDI and the mutant aggregate formation induced by SOD1(see Table 2) [191].

## 13 Multiple sclerosis (MS)

MS is a chronic neurological disorder in which myelinated exons of CNS started to degenerate by inflammation and autoimmune defects [192]. Epidemiological studies reveal that around 2.3 million people are suffering from this disease worldwide [193]. Environmental and genetic factors are responsible for the development of MS [194]. Role of NO in MS has been established but it is controversial in some cases [195]. Genetic studies have found the association between iNOS gene mutation and MS progression [196]. High level of iNOS RNA is found in the CNS of MS patients and animal models of MS [197]. Further, iNOS immunoreactivity has also been found in active lesions of MS patients compared to the normal human brain [198]. NO metabolites (nitrate and nitrite) level was increased in the CSF, urine and plasma of MS patients (see Fig. 4) [[199], [200], [201]]. These studies indicate that nitrite and nitrate can be used as potent markers for detection of MS. In some studies, nitrotyrosine has also been detected in MS patients [202,203]. Proteolipid protein (PLP) is responsible for maintenance and integrity of myelin sheath. Researchers hypothesized that S-nitrosylation of PLP (SNO-PLP) results in conformational and functional alterations of the protein, which lead to the disease progression (see Table 1) [195,204].

## 14 Conclusions

NO is one of the most important signalling molecules in the brain. It can play both protective/constitutive and destructive/toxic role, depending upon its regulation/production and interaction with different molecules in the cell. Therefore, NO is also referred to as a “double-edged sword”. A large body of the accumulated evidence suggests that NO is one of the key factors in the genesis of many brain-related disorders. Along with SNO, which targets a wide range of prominent intracellular proteins leading to alteration in signalling pathways, which may converge onto synaptic, neuronal and behavioral deficits. Although NO promotes neurogenesis, aberrant SNO may be responsible for different neurodevelopmental disorders, such as ASD. The SHANK3 study on NO implies it as an important pathological molecule in ASD. As neurons are probably the most vulnerable cell type in the body, oxidative and nitrosative stress and aberrant SNO play a role in the development of a variety of neuroinflammatory and neurodegenerative disorders, including PD, AD, HD, ALS and MS. Further, dysregulation of NO signalling can induce the development of different neuropsychiatric and neurological disorders, such as BPD, migraine, epilepsy, schizophrenia and addiction. Finally, pharmacological agents that can inhibit or augment the production of NO as well as new approaches to pharmacologically modulate formation of SNO-proteins can serve as a potential and promising approach for the treatment of diverse brain disorders.

## Declaration of competing interest

The authors have no competing interests to declare.
